# Broad-Spectrum HDAC Inhibitors Promote Autophagy through FOXO Transcription Factors in Neuroblastoma

**DOI:** 10.3390/cells10051001

**Published:** 2021-04-24

**Authors:** Katharina Körholz, Johannes Ridinger, Damir Krunic, Sara Najafi, Xenia F. Gerloff, Karen Frese, Benjamin Meder, Heike Peterziel, Silvia Vega-Rubin-de-Celis, Olaf Witt, Ina Oehme

**Affiliations:** 1 Hopp Children’s Cancer Center Heidelberg (KiTZ), 69120 Heidelberg, Germany; k.koerholz@kitz-heidelberg.de (K.K.); j.ridinger@kitz-heidelberg.de (J.R.); s.najafi@kitz-heidelberg.de (S.N.); xenia.gerloff@dkfz-heidelberg.de (X.F.G.); h.peterziel@kitz-heidelberg.de (H.P.); o.witt@kitz-heidelberg.de (O.W.); 2 Clinical Cooperation Unit Pediatric Oncology, German Cancer Research Center (DKFZ), German Cancer Research Consortium (DKTK), INF 280, 69120 Heidelberg, Germany; 3 Light Microscopy Facility (LMF), German Cancer Research Center (DKFZ), 69120 Heidelberg, Germany; D.Krunic@dkfz-heidelberg.de; 4 Department of Pediatric Oncology, Hematology and Immunology, University Hospital Heidelberg, 69120 Heidelberg, Germany; 5 Institute for Cardiomyopathies Heidelberg, Heidelberg University, 69120 Heidelberg, Germany; karen.frese@med.uni-heidelberg.de (K.F.); benjamin.meder@med.uni-heidelberg.de (B.M.); 6 Genome Technology Center, Stanford University, Stanford, CA 94304, USA; 7 Institute for Cell Biology (IFZ), University Hospital Essen, 45122 Essen, Germany; silvia.vega.rubindecelis@gmail.com

**Keywords:** macroautophagy, FOXO1, FOXO3a, vorinostat, panobinostat, neuroblastoma

## Abstract

Depending on context and tumor stage, deregulation of autophagy can either suppress tumorigenesis or promote chemoresistance and tumor survival. Histone deacetylases (HDACs) can modulate autophagy; however, the exact mechanisms are not fully understood. Here, we analyze the effects of the broad-spectrum HDAC inhibitors (HDACi) panobinostat and vorinostat on the transcriptional regulation of autophagy with respect to autophagy transcription factor activity (Transcription factor EB—TFEB, forkhead boxO—FOXO) and autophagic flux in neuroblastoma cells. In combination with the late-stage autophagic flux inhibitor bafilomycin A1, HDACis increase the number of autophagic vesicles, indicating an increase in autophagic flux. Both HDACi induce nuclear translocation of the transcription factors FOXO1 and FOXO3a, but not TFEB and promote the expression of pro-autophagic FOXO1/3a target genes. Moreover, FOXO1/3a knockdown experiments impaired HDACi treatment mediated expression of autophagy related genes. Combination of panobinostat with the lysosomal inhibitor chloroquine, which blocks autophagic flux, enhances neuroblastoma cell death in culture and hampers tumor growth in vivo in a neuroblastoma zebrafish xenograft model. In conclusion, our results indicate that pan-HDACi treatment induces autophagy in neuroblastoma at a transcriptional level. Combining HDACis with autophagy modulating drugs suppresses tumor growth of high-risk neuroblastoma cells. These experimental data provide novel insights for optimization of treatment strategies in neuroblastoma.

## 1. Introduction

Neuroblastoma is an embryonal tumor derived from progenitor cells of the sympathetic nervous system. It is the most common extracranial solid tumor in children and causes at least 10% of the pediatric cancer deaths [1,2]. Survival of neuroblastoma patients strongly depends on age at diagnosis, stage of disease and neuroblastoma tumor biology. Especially in high-risk patients, disease outcome is still poor and relapse rates are high underlining the need to develop novel therapeutic strategies and treatments (reviewed in [3,4]). 

Histone deacetylases (HDACs) catalyze the deacetylation of histones, as well as other nuclear and cytoplasmic proteins, including heat shock proteins, cytoskeletal proteins and transcription factors [5]. HDACs thus control pivotal cellular processes, such as DNA repair, protein folding, apoptosis and autophagy (reviewed in [6]) and HDACs are involved in various processes critical for tumorigenesis. HDACs have thus emerged as actionable targets in anti-cancer therapies (reviewed in [7]). Amongst others, their inhibition induces cell cycle arrest, differentiation and apoptosis. To date, four HDAC inhibitors (HDACis) have been approved by the FDA for clinical use in anti-cancer therapies, including vorinostat and panobinostat [8] (reviewed in [9]). Additionally, more than 20 HDACi are currently tested in clinical trials, either in single or combination treatment regimens (reviewed in [10,11,12]). 

Autophagy is a highly conserved degradation process by which cytoplasmic components such as dysfunctional organelles, misfolded proteins or invading microorganisms, are delivered to the lysosome for degradation. To maintain cellular homeostasis, autophagy occurs at basal level in all cells but can be upregulated in response to stress, such as nutrient deprivation or cytotoxic agents [13]. The autophagic process is regulated both by direct protein–protein interactions and also by transcription of autophagy and lysosome related genes. Pivotal transcription factors for these genes are TFEB (Transcription Factor EB), a member of the microphthalmia family of basic helix-loop-helix leucine-zipper (bHLH-Zip) transcription factor family (MiT/TFE family) and members of the FOXO (Forkhead box) transcription factor family [14,15,16] (reviewed in [17]). The activity of both TFEB and FOXO transcription factors is regulated by phosphorylation via mTORC1 (mechanistic target of rapamycin complex 1) and ERK2 (mitogen activated protein kinase 1, MAPK1/extracellular signal-related kinase 2, ERK2) for TFEB and AKT (protein kinase B, PKB/AK thymoma, AKT) for FOXO. When unphosphorylated, TFEB and FOXO transcription factors locate to the nucleus and initiate the transcription of target genes. Conversely, phosphorylation causes their sequestration in the cytoplasm and thus inactivates their transcriptional activity [18,19]. 

Deregulated autophagy is closely connected to tumorigenesis. Disruption of cell homeostasis by defective autophagy can increase DNA damage and inflammation and thereby promote early tumorigenesis. Conversely, in response to cellular stress such as anti-cancer therapy, established tumors can upregulate cytoprotective autophagy, which contributes to therapeutic resistance. Additionally, cancer progression and metastasis may be promoted by the pro-survival effects of autophagy [20,21,22]. Interference with autophagy has therefore emerged as promising cancer-directed therapeutic approach. 

Here, we investigate the impact of broad-spectrum HDACis on autophagic flux. We show that vorinostat and panobinostat induce autophagy, transcriptionally upregulate autophagy related genes in neuroblastoma cells and induce nuclear translocation of the autophagy transcription factors FOXO1 and FOXO3a. Combination of vorinostat or panobinostat with the autophagy modulating agent chloroquine enhances neuroblastoma cell death in cell culture and impairs tumor growth in vivo (zebrafish early larvae xenografts), thus evincing a great potential for combination treatment of autophagy competent tumors.

## 2. Materials and Methods

### 2.1. Cell Culture

Human neuroblastoma cell lines SK-N-BE(2)-C (European Collection of Authenticated Cell Cultures, ECACC, Salisbury, UK) and IMR-32 (German Collection of Microorganisms and Cell Cultures, DSMZ, Darmstadt, Germany) were cultured under standard conditions in Dulbecco’s Modified Eagles Medium (DMEM containing L-glutamine and 4.5 g/L glucose, Gibco Invitrogen cell culture, Invitrogen, Paisley, UK) supplemented with 10% fetal calf serum (FCS; Sigma, St. Louis, MO, USA) and 1% non-essential amino acids (NEAA; Invitrogen, Carlsbad, CA, USA). All cell lines were regularly checked for mycoplasma and multiple contaminations (Multiplexion, Heidelberg, Germany) and routinely verified using DNA fingerprinting authentication by Multiplexion.

### 2.2. Cell Culture Reagents

Vorinostat (Selleckchem, Houston, TX, USA, 1 M stock), panobinostat (Cayman- Biomol, Hamburg, Germany, 50 mM stock), bafilomycin A1 (Santa Cruz, Dallas, TX, USA, 10 µM stock), perifosine (Cayman—Biomol, 10 mM stock), rapamycin (Sigma-Aldrich, Munich, Germany, 1 mM stock), okadaic acid (Santa Cruz, Dallas, TX, USA, 1 mM stock) were dissolved in dimethyl sufoxide (DMSO). Chloroquine (CQ; Sigma-Aldrich, 100 mM stock) was dissolved in autoclaved Millipore H_2_O. If not otherwise specified, compounds were stored at −20 °C (vorinostat was stored at −80 °C) and protected from light. 

### 2.3. siRNA Transfection

Cells were transfected with siRNA using QIAGEN HiPerFect transfection reagent. Cells were seeded at a density of 4 × 10^5^ cells per 10 cm dish and transfected the next day. Transfection mixes were prepared in sterile 1.5 mL reaction tubes as follows: 50 µL of siRNA (2 µM) were added to 20 µL HiPerFect followed by 930 µL of OptiMEM to yield a siRNA concentration of 100 nM. Transfection mixes (1 mL) were added to 10 cm dishes containing 3 mL of cell culture media after 10 min of incubation at room temperature (final siRNA concentration: 25 nM). A pool of three siRNAs was used for FOXO1 (Ambion s5257, s5258, s5259) and FOXO3a (Ambion s5260, s5262, s5261) knockdown. Negative control transfection was performed with a pool of two siRNAs (Silencer Negative Control #1 and Silencer Negative Control #5, Ambion AM4611, AM4642). Medium was changed 16–18 h post transfection

### 2.4. Western Blot Analysis

Cells were seeded on 10 cm dishes and treated for 24 h. Cells were lysed in sodium dodecyl sulfate (SDS) lysis buffer (Tris 0.5 M, hydrochloric acid (HCl) (pH6.8), SDS 2%, 87%-glycerol 10%, dithiothreitol (DTT) 1 mM). Protein samples were denatured at 95 °C for 10 min. Bromophenol blue stock solution was added to 30 µg protein (15 µg for LC3B detection) and loaded to SDS-polyacrylamide gels. Proteins were subjected to electrophoretic separation, followed by blotting to polyvinylidene fluoride (PVDF) membranes using a semidry electroblot chamber (blotting time according to the size of the protein of interest). Transfer of the proteins was assessed by ponceau-red staining. Membranes were blocked in blocking solution (Tris-buffered saline, Nonfat dry milk 20%, FCS 20%, bovine serum albumin (BSA) 3%, normal goat serum (NGS) 1%, Tween20 0.2%) for 1 h. Incubation with a primary antibody solution over night was performed at 4 °C followed by 1 h incubation with a peroxidase-conjugated secondary antibody at room temperature. The following antibodies were used for detection by the electrogenerated chemiluminescence (ECL) method: anti-LC3B (L7543, Sigma, Munich, Germany), anti-phosphorylated AKT (Ser473) (9271, Cell Signaling Technology, Cambridge, UK), anti-AKT (9272, Cell Signaling Technology), anti-phosphorylated FOXO1 (Ser256) (9461, Cell Signaling Technology), anti-FOXO1 (2880, Cell Signaling Technology), anti-FOXO3a (2497s, Cell Signaling Technology), anti-phosphorylated ERK1/2 (Thr202/Tyr204) (4370S, Cell Signaling Technology), anti-ERK1/2 (4695S, Cell Signaling Technology), anti-TFEB (4240, Cell Signaling Technology), anti-phosphorylated S6K1 (Thr389) (07018SP, Millipore, Billerica, MA, USA), anti-S6K1 (9202S, Cell Signaling Technology), anti-HDAC2 (sc-81599, Santa Cruz Biotechnology, Dallas, TX, USA), anti-p62 (P0067, Sigma), anti-GAPDH (Lot Number JC1682928, Millipore, Burlington, MA USA) and anti-β-actin (clone AC-15; Sigma). Quantification of Western Blot images was performed using ImageJ-software version 1.0.

### 2.5. Subcellular Fractionation Analysis

Cells were seeded on 10 cm dishes and treated for 24 h. Cell lysates were prepared with the Nuclear/Cytosol Fractionation Kit (BioVision, Milpitas, CA, USA) according to the manufacturer’s instructions. Western blot analysis was performed as described above. 

### 2.6. Real-Time PCR Analysis

Cells were seeded in 6-well plates and treated for 24 h. Cells were lysed using RLT-buffer (RNeasy Protect Mini Kit, QIAGEN, Hilden, Germany) supplemented with 2-mercaptoethanol. Lysates were frozen at −80 °C overnight, diluted with an equal volume of 70% ethanol after rethawing and RNA was isolated using the RNeasy Mini Kit according to the manufacturer’s instructions. One µg of RNA was reversely transcribed into cDNA using RevertAid First Strand cDNA-Synthesis Kit (ThermoFisher Scientific, Braunschweig, Germany) according to the manufacturer’s instructions. mRNA expression in cells was quantified by real-time reverse transcription PCR with qPCR MasterMix for SYBR^®^ Green 1 (Eurogentec, Kaneka Eurogentec S.A., Seraing, Belgium). Unless otherwise specified, primers were purchased from ThermoFischer Scientific. The following primers were used: *FOXO1* (forward: 5′-AAC CTG GCA TTA CAG TTG GCC-3′, reverse: 5′-AAA TGC AGG AGG CAT GAC TAC GT-3′), *FOXO3a* (forward: 5′-GGG ACA AAC GGC TCA CTC T-3′, reverse: 5′-GGA CCC GCA TGA ATC GAC TAT-3′), *GABARAPL1* (forward: 5′-ATG AAG TTC CAG TAC AAG GAG GA-3′, reverse: 5′-GCT TTT GGA GCC TTC TCT ACA AT-3′), *MAP1LC3a* (forward: 5′-AAC ATG AGC GAG TTG GTC AAG-3′, reverse: 5′-GCT CGT AGA TGT CCG CGA T-3′), *WIPI1* (forward: 5′-AGT CAG TCA CAC AAA ACC ACG-3′, reverse: 5′-AGA GCA CAT AGA CCT GTT GGG-3′), *BNIP3L* (forward: 5′-ATG TCG TCC CAC CTA GTC GAG-3′, reverse: 5′-TGA GGA TGG TAC GTG TTC CAG-3′), *HPRT* (forward: 5′-TGA CAC TGG CAA AAC AAT GCA-3′, reverse: 5′-GGT CCT TTT CAC CAG CAA GCT-3′), *SDHA* (forward: 5′-TGG GAA CAA GAG GGC ATC TG-3′, reverse: 5′-CCA CCA CTG CAT CAA ATT CAT G-3′). Data were expressed as relative gene expression (fold change) according to the 2−ΔΔCt method [23], normalized to neuroblastoma housekeeping genes *SDHA* and *HPRT* [24] and set in relation to negative control.

### 2.7. Quantification of Microscopic Images

Confocal microscopy images were quantified with ImageJ software version 1.0, using an in-house programmed semi-automated image analysis macro (source code in Supplementary Materials). Nuclei were counted to assess cell number and green as well as red fluorescent vesicles were determined. 

### 2.8. Cell Viability Assays

Adherent cell lines were detached using Trypsin-ethylenediaminetetraacetic acid (Trypsin-EDTA; Thermo Fischer Scientific) and cells were pooled with corresponding supernatant, centrifuged and resuspended in 1 mL complete medium. Cell viability was measured by automated trypan blue staining using the Vi-Cell XR Cell Viability Analyzer (Beckman Coulter, Krefeld, Germany) with three technical replicates per treatment and at least three independent experiments. For one technical replicate 50 images were generated and living as well as dead cells (trypan blue positive) were automatically counted.

### 2.9. Colony Formation Assays

Cells were seeded on six-well plates at a density of 2000 cells per well and treated for 24 h. After medium change adherent cells were cultured for 11 additional days before staining of viable cell colonies with 1%-crystal violet staining solution. Quantification was performed using ImageJ Fiji version 2.1.0, applying the ITCN plugin.

### 2.10. Zebrafish Lines

Care and breeding of zebrafish were done under standardized conditions and as described previously [25]. Zebrafish wild-type AB line was raised at 28 °C. Embryos used for tumor injections were maintained in E3 buffer supplemented with 0.2 mM 1-phenyl-2-thiourea (PTU, Sigma). Zebrafish husbandry and experiments were performed according to local animal welfare standards (Tierschutzgesetz §11, Abs. 1, No. 1) and in accordance with European Union animal welfare guidelines (EU Directive 2010/63/EU). All applicable national and institutional guidelines for the care and use of zebrafish were followed. All procedures performed involving animals were in accordance with ethical standards of the institution.

### 2.11. Cell Preparation and Zebrafish Larvae Xenotransplantation

SK-N-BE(2)-C cells were cultured to 70–80% confluence, then washed once with phosphate-buffered saline (PBS; Lonza, Basel, Switzerland), trypsinized (Gibco), counted and resuspended in phenol red-free Roswell Park Memorial Institute medium (RPMI, Gibco). Tumor cells were labeled as described previously [25]. Briefly, cells were incubated with CellTracker CM-DiI (Thermo Fisher Scientific, Braunschweig, Germany Waltham, MA, USA) for 5 min at 37 °C and then for an additional 15 min at 4 °C. To minimize cell clumping, DNase I (250 Kunitz units/mL, Sigma) was added to the cell suspension. Following the incubation, cells were washed with 10% FCS RPMI, twice with serum-free RPMI and resuspended in serum-free RPMI to a final concentration of 1.0 × 10^8^ cell/mL. Before implantation, zebrafish were anesthetized with tricaine (0.02%, Sigma) and embedded in a lateral position in 1.0% low gelling temperature agarose (Sigma). Between 150 and 250 CM-DiI-labeled tumor cells were injected into the yolk sac of each zebrafish embryo using FemtoJet express microinjector (Eppendorf, Hamburg, Germany) and glass microinjection needles (Science Products, Hofheim, Germany). Embryo were transferred to 34 °C 1 h after tumor cell injection.

### 2.12. Zebrafish Larvae Drug Treatment and Efficiency Evaluation

Treatment was performed as described previously [25]. Briefly, tumor xenografts were evaluated by fluorescence microscopy (Olympus, Hamburg, Germany) 2 h post implantation. Only larvae with red fluorescence at the injection site were used for drug testing. Selected embryos were transferred to 48-well uncoated plates (Corning) and incubated in PTU buffer containing drugs or solvent. The medium was replaced daily. Tumor growth was evaluated by confocal microscopy before drug exposure as well as 48 h post treatment. For imaging, fish were anesthetized with tricaine (0.02%, Sigma) and embedded in a lateral position in 1.0% low gelling temperature agarose (Sigma) in chambered coverslips (Ibidi, Martinsried, Germany). Images of living early larvae were obtained using a Zeiss LSM 710 confocal microscope (Zeiss, Oberkochen, Germany) and ZEN software (Zeiss). Tumor progression was evaluated using Fiji software and a semi-automated macro [25]. Zebrafish embryos were treated with higher concentrations of panobinostat (200 nM) and chloroquine (100 µM) as substances are applied to the surrounding water and, as the estimated extent of compound absorption by the zebrafish early larvae is one-tenth to one-twentieth of the cell culture treatment concentration [26,27].The maximal tolerated dose for chloroquine was above 500 µM and for panobinostat 1 µM, as described previously [25]. 

### 2.13. Gene Expression Analysis

For microarray analysis, three independent replicate experiments were performed, in which SK-N-BE(2)-C cells were treated for 24 h with 500 nM vorinostat and solvent (DMSO), respectively. Total RNA was isolated using the RNeasy MiniKit (Qiagen). For microarray analysis, 1 µg RNA per sample was used. Gene expression analysis was performed at the house-internal Genomics and Proteomics Core Facility using human whole genome HT-12 v4 BeadChips^®^. Normalization of the raw intensity data was performed by the microarray unit of the DKFZ Genomics and Proteomics Core Facility with Illumina BeadStudio Data Analysis Software version v4_r2. GEO number: GSE169219. The normalized gene expression profiles were further analyzed by R2 (R2: microarray and visualization platform: http://r2.amc.nl, accessed on 16 March 2021). R2 was used to find differentially expressed autophagy-related genes (GO: 0006914) between solvent and vorinostat treated SK-N-BE(2)-C cells. Significantly (*p* < 0.05) (analysis of variance (ANOVA), corrected for multiple testing) differentially expressed autophagy-related genes were depicted as heatmap.

### 2.14. Statistical Analysis

R (R version 4.0.3, 2020; The R Foundation for Statistical Computing) and R package CRAN ggplot2 and RColorBrewer [28,29] was used for statistical analysis. Data were analyzed using two-tailed *t*-test or one-way analysis of variance (ANOVA). For multiple comparisons, Tukey’s post hoc corrections were applied. A *p*-value of *p* < 0.05 was considered significant. The number of replicates for each experiment is depicted in the respective figure or figure legend. 

## 3. Results

### 3.1. Broad-Spectrum HDAC Inhibitors Induce Autophagic Flux in SK-N-BE(2)-C Neuroblastoma Cells 

**Figure 1:**
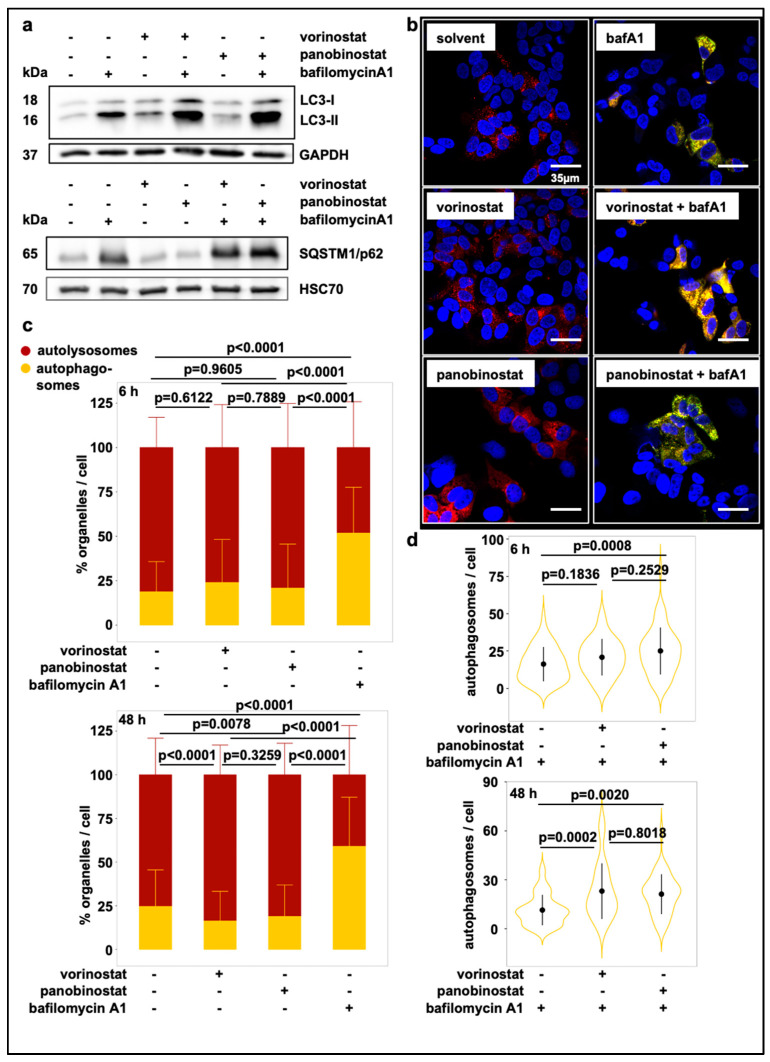
Treatment with broad-spectrum HDAC inhibitors induces autophagic flux. (**a**) Western Blot displaying LC3-I and LC3-II levels and SQSTM1/p62 levels in whole-cell lysates of SK-N-BE(2)-C cells after 24 h treatment with 500 nM vorinostat or 10 nM panobinostat or solvent control and with or without additional 6h treatment with bafilomycin A1 (100 nM). GAPDH and HSC70 served as a loading control for corresponding Western blots. (**b**) Confocal fluorescence microscopy analysis of SK-N-BE(2)-C cells. Autophagosome formation was visualized after treatment with vorinostat (500 nM) or panobinostat (10 nM) alone or in combination with 100 nM bafilomycin A1 by using the mCherry-EGFP-LC3B expression construct. Autolysosomes appear red fluorescent only, autophagosomes are red and green fluorescent (yellow in merged channels). Scale bar: 35 µm. (**c**) Automated quantification of one representative fluorescence microscopic experiments using the mCherry-EGFP-LC3B expression construct 6 h and 48 h after treatment with either HDACi or bafilomycin A1. Each transfected cell was analyzed for green and red fluorescent foci. Statistical analyses: ANOVA with Tukey’s multiple comparison test. (**d**) Automated quantification and statistical analyses for the combination treatment with bafilomycin A1 as described in (**c**).

Broad-spectrum HDAC inhibitors show promising anti-cancer activities in several tumor entities, including neuroblastomas. To investigate whether and how HDAC inhibitors affect autophagy in neuroblastoma, we analyzed autophagic flux in SK-N-BE(2)-C cells upon treatment with two widely used broad-spectrum HDAC inhibitors, vorinostat and panobinostat. We selected SK-N-BE(2)-C cells, which are derived from a relapsed patient after chemotherapy [30] and recapitulate many aspects of advanced stage neuroblastoma, such as *TP53* (tumor protein p53) mutation, *MYCN* (v-myc avian myelocytomatosis viral oncogene neuroblastoma derived homolog) amplification and relative treatment resistance towards chemotherapeutics [31]. Nonetheless, these cells are still responsive to HDAC inhibitor treatment [32]. To detect autophagic flux, we assessed the amount of phosphatidylethanolamine conjugated microtubule-associated protein light chain 3 (LC3-II), an autophagy marker localized to the autophagosome membrane, after treatment with either HDAC inhibitor alone or in combination with the late stage autophagy inhibitor bafilomycin A1 [33]. Bafilomycin A1 interferes with the lysosomal V-ATPase thereby inhibiting lysosomal acidification and preventing degradation of engulfed contents [34]. The treatment of SK-N-BE(2)-C cells with either HDAC inhibitor alone appeared to increase the amount of conjugated LC3-II and appeared to decreased the amount of sequestome-1 (p62/SQSTM1) compared to the solvent control, suggesting that the treatment stimulated autophagic flux (Figure 1a; Supplementary Figure S1a,b). If so, the combination of HDAC inhibitor with the late stage autophagy inhibitor bafilomycin should further increase accumulation of autophagosomes when compared to bafilomycin treatment alone, since de novo generated autophagosomes accumulate at the stage of fusion with the lysosome. Hence, we added bafilomycin A1 for 6h to the HDAC inhibitor treatment. The combination treatment appeared to increase the levels of conjugated LC3-II as well as p62/SQSTM1 further, pointing towards autophagic flux induction through HDAC inhibitor treatment, which, if late stage fusion is blocked, enhances accumulation of autophagosomes albeit the effect did not reach statistical significance over three experimental replicates (Figure 1a; Supplementary Figure S1a,b). To confirm with a second, statistically more robust, method that treatment of SK-N-BE(2)-C cells with vorinostat and panobinostat promotes autophagic flux, we used cells stably expressing a tandem EGFP-mCherry tagged LC3B and conducted automated microscopic experiments, followed by automated image analysis (using an in-house programmed ImageJ Macro) (see Supplementary Methods). Features extracted from automated image analysis included cell number by counting of DAPI stained nuclei, red fluorescent vesicles and vesicles with red and green double-fluorescence. Vesicles with red and green fluorescence overlay represent autophagosomes. Upon fusion with lysosomes they lose their EGFP fluorescence due to quenching [35], making autolysosomes appear red fluorescent only. Therefore, increased solely red fluorescent vesicle numbers indicate increased number of autolysosomes (pointing to increased autophagic flux), whereas an increase of vesicles with red and green overlay indicates impaired fusion of autophagosomes and lysosomes (blocked autophagic flux). We used two different time points (6 h and 48 h) to get a hint whether HDAC inhibitors vorinostat and panobinostat directly affected the autophagic machinery (6 h time point) or whether the observed effects on autophagic flux might depend on gene transcription (48 h time point). Treatment of SK-N-BE(2)-C cells with vorinostat or panobinostat significantly increased the relative number of autolysosomes after 48h treatment, while decreasing the relative number autophagosomes (Figure 1b,c; Supplementary Figures S1c and S2). This supports our finding that vorinostat and panobinostat promote autophagic flux. In contrast, bafilomycin A1, which blocks fusions of autophagosomes with lysosomes, increased relative autophagosome numbers while decreasing autolysosome numbers after 6h and 48h, respectively (Figure 1b,c). Due to their autophagic flux-inducing effect, vorinostat or panobinostat should increase relative autophagosome numbers upon co-treatment with a late-stage flux inhibitor such as bafilomycin A1, because de-novo generated autophagosomes are unable to fuse with lysosomes. We observed that co-treatment with bafilomycin and vorinostat or panobinostat for 48h significantly increased autophagosome numbers when compared to bafilomycin treatment alone (Figure 1b,d, Supplementary Figures S1c and S2), confirming that vorinostat and panobinostat promote autophagy in SK-N-BE(2)-C cells.

### 3.2. Broad-Spectrum HDAC Inhibitors Increase the Expression of Autophagy Genes in Neuroblastoma Cell Lines

**Figure 2:**
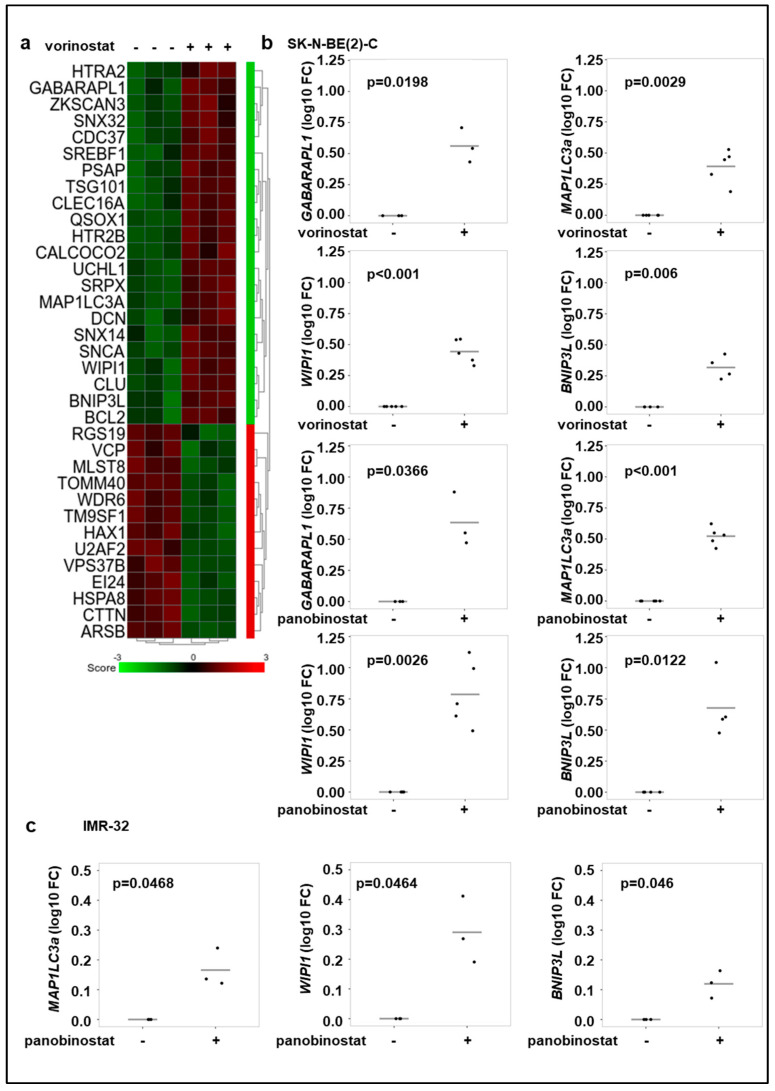
Broad-spectrum HDAC inhibitor treatment increases the expression of autophagy genes. (**a**) Gene expression analysis of genes with the ontology term “autophagy” of SK-N-BE(2)-C neuroblastoma cells. Cells were treated for 24 h with vorinostat (500 nM) in three replicates. Genes, which are significantly up- or downregulated (*p* < 0.05) are shown. (**b**) Realtime RT-PCR analysis of *GABARAPL1*, *MAP1LC3a*, *WIPI1*, *BNIP3L* after 24 h treatment of SK-N-BE(2)-C cells with vorinostat (500 nM) or panobinostat (10 nM). (**c**) Realtime Quantitative RT-PCR analysis of *MAP1LC3a*, *WIPI1*, *BNIP3L* after 24 h treatment of IMR-32 cells with panobinostat (4 nM). Statistical analyses were performed using one sample *t*-test. Boxes represent the SD.

As vorinostat and panobinostat promote autophagic flux and are well-known modulators of gene transcription, we investigated the effect of these HDAC inhibitors on the expression of autophagy-related genes. The microarray expression analysis of genes with the ontology term “autophagy” (GO:0006914) revealed a significant upregulation of several autophagy genes in SK-N-BE(2)-C cells treated with vorinostat for 24 h (Figure 2a). Realtime RT-PCR analyses confirmed these findings for some selected genes (*GABARAPL1*, *MAP1LC3a*, *WIPI1* and *BNIP3L)*, as treatment with the both broad-spectrum HDAC inhibitors, vorinostat or panobinostat, resulted in upregulation of these genes in SK-N-BE(2)-C and IMR-32 neuroblastoma cells (Figure 2b,c). Of note, *GABARAPL1* was not detectable in IMR-32 cells.

### 3.3. Broad-Spectrum HDAC Inhibitors Do Not Reduce mTORC1 Activity or Induce TFEB Translocation, But Activate FOXO1 and FOXO3 Transcription Factors in SK-N-BE(2)-C Cells 

**Figure 3:**
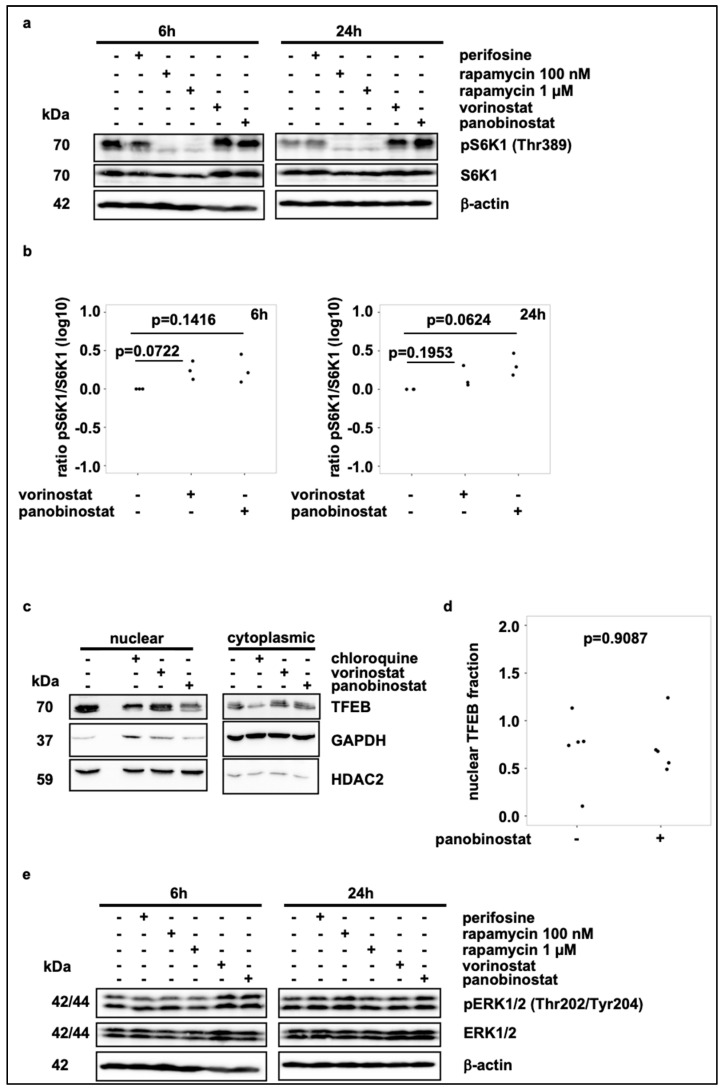
Broad-spectrum HDAC inhibitor treatment does not reduce mTORC1 activity. (**a**) Western blot analysis of pS6K1 and S6K1 levels after 6 h and 24 h treatment with vorinostat (500 nM) or panobinostat (10 nM). Rapamycin (100 nM and 1 µM) was used as negative control for mTORC1 activity and thus S6K1 phosphorylation. Note that blots were also used to analyze ERK1/2 phosphorylation in Figure 3e. (**b**) Quantification of Western blot analysis of pS6K1 and S6K1 expression, normalized to solvent control. Statistical analyses: ANOVA with Tukey’s multiple comparison test. (**c**) Subcellular localization analysis of TFEB on SK-N-BE(2)-C cells treated for 24 h with vorinostat (500 nM) or panobinostat (10 nM). Chloroquine (50 µM) was used as positive control for TFEB translocation. GAPDH was used as cytoplasmic marker, HDAC2 was used as nuclear marker. (**d**) Quantification of nuclear TFEB by Western blot analysis from subcellular localization analysis. Statistical analyses: *t*-test. (**e**) Western blot analysis of pERK1/2 and ERK1/2 expression after 6 h and 24 h treatment with vorinostat (500 nM) or panobinostat (10 nM). Note that identical blots were used to analyze S6K1 phosphorylation in Figure 3a.

The mechanistic target of rapamycin complex 1 (mTORC1) is known to be a key regulator in the autophagy process. Increased mTORC1 activity is known to suppress catabolic processes, such as autophagy via the Unc-51-like autophagy activating kinase-1 (ULK1) and anabolic programs are induced by increased mTORC1 activity via phosphorylation of the ribosomal protein S6 kinase beta-1 (S6K1) (reviewed in [36]). Hence, the phosphorylation state of S6K1 is a surrogate marker for mTORC1 activity. We investigated whether vorinostat and panobinostat treatment induces autophagy in SK-N-BE(2)-C cells by reducing mTORC1 activity. Treatment of SK-N-BE(2)-C cells with vorinostat or panobinostat for 24 h did not diminish, but rather induced phosphorylation of S6K1 (Figure 3a,b), which is indicative for activated mTORC1. In contrast, the positive control rapamycin, a well-known mTOR inhibitor, markedly reduced phosphorylation of S6K1 (Figure 3a). We conclude, that the HDAC inhibitor initiated autophagic flux is not mediated via mTOR inactivation.

As HDAC inhibitor treatment upregulates the expression of ATG genes, we wondered whether HDAC inhibitor treatment of SK-N-BE(2)-C cells affects the transcriptional regulation of these genes. Thus, we investigated the activity of the transcription factors TFEB, FOXO1 and FOXO3, which are known to regulate the expression of autophagy and lysosomal related genes (reviewed in [17]).

The activity of TFEB and the FOXO family members FOXO3a and FOXO1 is regulated by phosphorylation, such that they only locate to the nucleus and activate target genes transcription in an unphosphorylated state. Conversely, phosphorylation via ERK2 and mTORC1 (TFEB) or via the PI3K-AKT pathway (FOXO) causes their retention in the cytoplasm [18,19]. Neither treatment with vorinostat nor panobinostat affected nuclear translocation of TFEB in SK-B-BE(2)-C cells (Figure 3c,d; Supplementary Figure S3a). Moreover, neither vorinostat nor panobinostat treatment affected ERK phosphorylation in SK-N-BE(2)-C cells (Figure 3e; Supplementary Figure S3b). 

**Figure 4:**
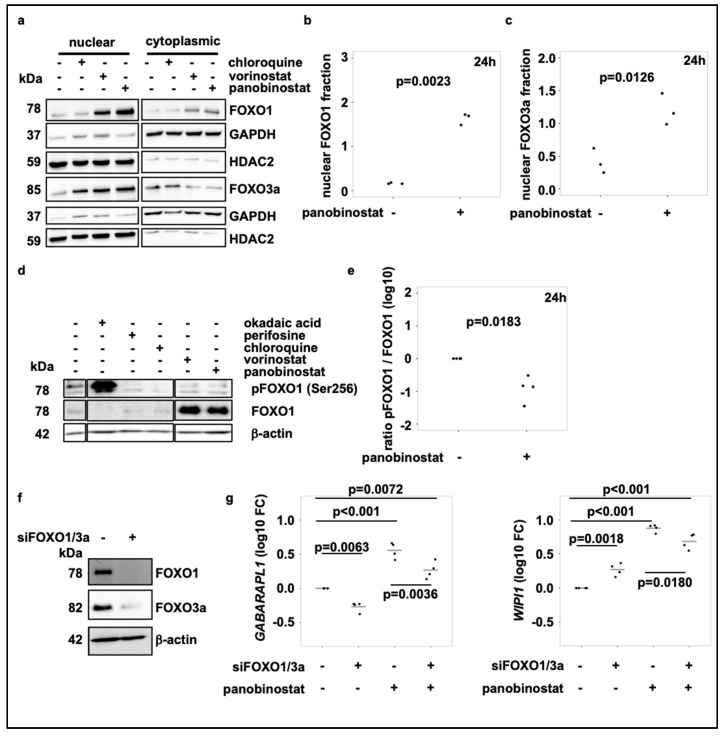
Broad-spectrum HDAC inhibitor treatment activates FOXO1 via the PI3K-AKT-pathway and induces FOXO nuclear translocation. (**a**) Representative subcellular localization analysis of FOXO1 and FOXO3a in SK-N-BE(2)-C neuroblastoma cells treated for 24 h with chloroquine (50 µM), vorinostat (500 nM) or panobinostat (10 nM) for 24 h. GAPDH was used as cytoplasmic marker, HDAC2 served as nuclear marker. (**b**,**c**) Quantification of FOXO1 (**b**) and FOXO3a (**c**) nuclear protein of at least three individual Western blots analogous to figure (**a**). (**d**) Western blot analysis of pFOXO1 and FOXO expression after treatment of SK-N-BE(2)-C cells with chloroquine (50 µM), vorinostat (500 nM) or panobinostat (10 nM) for 24 h. Okadaic acid (1 µM) and perifosine (5 µM) were used as positive and negative control, respectively. All samples were applied to the same gel and blotted on one membrane. Treatments with inhibitors/substances not relevant for the figure were cut from the analysis. (**e**) Quantification of the pFOXO1 to FOXO1 ratio from Western blot analysis from four individual experiments, normalized to solvent control. Statistical analyses: *t*-test. (**f**) Western blot analysis of FOXO1 and FOXO3a in SK-N-BE(2)-C neuroblastoma cells 5 days after transfection with control or FOXO1 and FOXO3a siRNA (pool of three different siRNAs for each target; pool of 6 siRNAs in total), respectively. (**g**) Realtime RT-PCR analysis of *GABARAPL1* and *WIPI1* after transfection of SK-N-BE(2)-C neuroblastoma cells with control or FOXO1 and FOXO3a siRNA (pool of 3 siRNAs for each target; pool of 6 siRNAs in total). Cells were additionally treated for the last 24h with either panobinostat or a solvent control. Statistical analyses: ANOVA with Tukey’s multiple comparison test.

However, treatment of SK-N-BE(2)-C cells with vorinostat or panobinostat substantially increased nuclear levels of FOXO1 and FOXO3a compared to solvent control (Figure 4a–c; Supplementary Figure S3c,d). In line with this, vorinostat and panobinostat treatment reduced the phosphorylation of FOXO1. Of note, both HDAC inhibitors markedly increased the protein levels of FOXO1 (Figure 4d). Vorinostat or panobinostat treatment decreased the ratio of phosphorylated FOXO1 to total FOXO1 five- to ten-fold (Figure 4e; Supplementary Figure S3e). Hence, treatment of SK-N-BE(2)-C cells with vorinostat and panobinostat induced nuclear translocation and activation of FOXO1 and FOXO3a transcription factors. Moreover, double knockdown of FOXO1 and FOXO3a significantly reduced HDACi induced upregulation of autophagy genes *WIPI1* and *GABARAPL1* (Figure 4f,g; Supplementary Figures S3f and S4).

### 3.4. Treatment of SK-N-BE(2)-C Cells with Panobinostat in Combination with Chloroquine Reduces Cell Viability and Tumor Growth in Cell Culture and In Vivo

**Figure 5:**
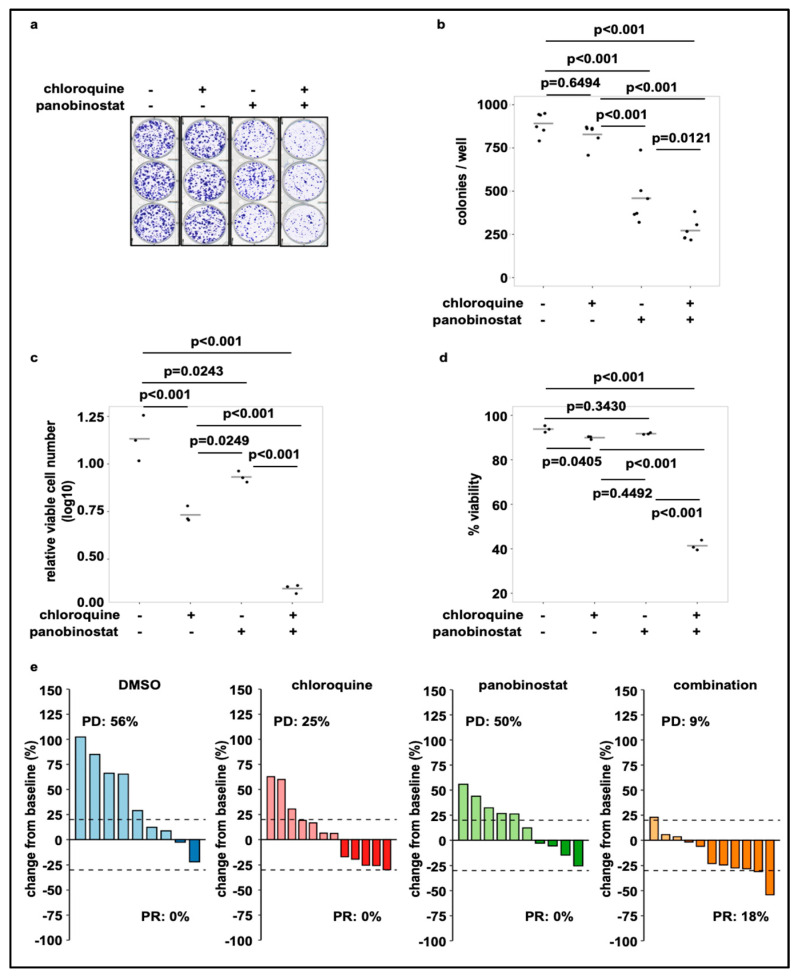
Treatment with panobinostat in combination with chloroquine reduces cell viability and colony growth. (**a**) Colony formation assay of SK-N-BE(2)-C neuroblastoma cells treated for 24 h with chloroquine (20 µM) or panobinostat (10 nM) alone or in combination. Shown are 6 technical replicates from two independent experiments. (**b**) Quantification of grown colonies after treatment of SK-N-BE(2)-C cells for 24 h with chloroquine (20 µM) or panobinostat (10 nM) alone or in combination. (**c**) Viable cell number (normalized to DMSO) of SK-N-BE(2)-C cells after 72 h treatment with chloroquine (20 µM) or panobinostat (10 nM) alone and in combination. (**d**) Viability of SK-N-BE(2)-C cells after 72 h treatment with chloroquine (20 µM) or panobinostat (10 nM) alone and in combination. (**b**–**d**) Statistical analyses: ANOVA with Tukey’s multiple comparison test. (**e**) Waterfall plots demonstrating change in tumor volume (%) for each individual xenograft, from baseline (day 1 = start of the treatment) to day 3 after yolk sac-implantation of SK-N-BE(2)-C cells. Dotted lines are drawn according to Response Evaluation Criteria in Solid Tumors (RECIST) 1.1 adopted for zebrafish tumors, to visualize best response: progressive disease (PD), at least a 20% increase in tumor volume; partial response (PR), at least a 30% decrease in tumor volume; each bar reflects one individual xenograft. The following concentrations were applied: chloroquine: 100 µM; panobinostat: 200 nM.

HDACi mediated induction of autophagy has been linked to therapeutic resistance to HDACi treatment [37] and could be disrupted by combining HDACi with autophagy inhibitors, such as chloroquine. Additionally, cell death induction by HDACi treatment was enhanced by combination treatment with autophagy inhibiting drugs [38,39]. To investigate the interplay of autophagic flux inducing broad-spectrum HDAC inhibitors with autophagy inhibiting drugs in our neuroblastoma cell model, we performed neuroblastoma colony assays. Co-treatment of SK-N-BE(2)-C cells with chloroquine and panobinostat effectively reduced colony formation (Figure 5a,b). In line with blocked colony formation, the number of viable cells (Figure 5c) and cell viability (Figure 5d) were substantially reduced by combination treatment with chloroquine and panobinostat, indicating a beneficial effect of the combination. To investigate treatment efficacy in vivo we used a zebrafish embryo/early larvae SK-N-BE(2)-C xenograft model. We tested panobinostat and chloroquine alone and in combination. Data are depicted as waterfall plots illustrating the change in tumor volume over baseline for the individual fish larvae (Figure 5d). Treatment with the combination of chloroquine and panobinostat substantially improved the response rate (PR = partial response according to RECIST, adopted for zebrafish) [25] with 0% PR for panobinostat or chloroquine alone and 18% for the combination. The progression rate (PD = progressive disease) decreased from 25% (chloroquine) and 50% (panobinostat) for the single treatment to 9% for the combination (Figure 5d).

## 4. Discussion

Despite extensive and multimodal therapy regimens relapse occurs frequently in high-risk neuroblastoma patients, with relapsed tumors being highly therapy resistant [40]. Autophagy is closely linked to chemoresistance in various cancer entities, including neuroblastoma [41,42,43]. Targeting autophagy in neuroblastoma therefore represents a promising approach to overcome treatment resistance and improve outcome of high-risk patients. 

Due to their involvement in numerous cancer-regulating processes, HDACs have emerged as potential target for cancer directed therapies. Given their good actionability, multiple HDAC inhibitors have been approved for cancer treatment or are currently tested in clinical trials (reviewed in [44]). We have previously identified HDAC10 as a poor prognostic marker in high-risk neuroblastoma, where it promotes chemoresistance via autophagy, lysosomal exocytosis and DNA double-strand break repair [45,46]. Interference with autophagy therefore represents one potential mechanism how HDAC inhibitors can impede tumor resistance mechanisms. Apart from HDAC10, there are other HDACs, which regulate autophagy, but their individual impact is diverse and partially unsolved. Consequently, a better understanding of (transcriptional) autophagy regulation in response to epigenetic therapy is still required.

In this study, we investigated the regulation of autophagy upon broad-spectrum HDACi treatment in neuroblastoma cells. Vorinostat and panobinostat increased autophagic flux and gene expression analysis revealed upregulation of numerous autophagy genes after HDACi treatment. Autophagy induction by HDACi treatment has been previously described in other cancer models such as HeLa cells [47] or malignant rhabdoid tumor cells [48], where autophagy was induced alongside of apoptotic cell death. HDACi can promote autophagy through a variety of mechanisms, including p53 signaling [49], ROS accumulation [50], p21 upregulation [51] or changes in mTOR signaling [52]. Moreover, autophagy is regulated at a transcriptional level by several transcription factors such as TFEB and members of the FOXO family. In our study, treatment of neuroblastoma cells with broad-spectrum HDACi vorinostat and panobinostat promoted autophagy via activation of FOXO1 and FOXO3a. We demonstrate that treatment with panobinostat and vorinostat induced nuclear translocation of FOXO1 and FOXO3a, while the simultaneous knockdown of FOXO1 and FOXO3a suppressed induction of autophagy genes, such as *WIPI1* and *GABARAPL1* [53,54] after HDACi treatment. Of note, simultaneous knockdown of FOXO1 and FOXO3a alone induced *WIPI1* expression, pointing towards an alternative regulation under these conditions, e.g., through TFEB [55] or TFE3 [56]. Our findings are supported by work of Zhang et al., which demonstrated enhanced FOXO1 transcriptional activity after trichostatin A (TSA) treatment and that knockdown or inhibition of FOXO1 blocked HDACi-mediated autophagy in colon cancer cells [39]. Furthermore, Pei et al. observed strong HDACi induced FOXO1 upregulation in FOXO1 low expressing *MYC*amp medulloblastoma cells [57]. In contrast to FOXO1 and FOXO3a transcription factor activation, we did not find alterations in mTOR signaling, one of the best described mechanisms of autophagy regulation, after vorinostat and panobinostat treatment, (reviewed in [58]). mTOR is regulated via the PI3K-AKT pathway, as well as AMPK (AMP-activated protein kinase) and its activation suppresses autophagy by inactivation of the ULK1 kinase. Earlier studies have shown that vorinostat inactivates mTOR in mouse embryonic fibroblasts (MEFs), relieving ULK1 from inactivation by mTORC1 and therefore inducing autophagy [52]. In contrast, two studies using leukemic cells showed that HDACi treatment can also suppress autophagy by transcriptional and post-translational repression of ATG7 [59] and activation of the PI3K/AKT/mTOR axis, respectively [60]. 

Moreover, we did not observe nuclear translocation of TFEB, a master transcription factor of autophagy and lysosomal genes, after broad-spectrum HDACi treatment of neuroblastoma cells. This is in line with our observation that panobinostat and vorinostat treatment did not alter mTOR activity, as TFEB localization is controlled by phosphorylation via mTORC1 such that mTORC1 activity prevents nuclear translocation of TFEB [61]. TFEB phosphorylation at S142 is additionally controlled by ERK2 [18]. Consistent with the lack of TFEB translocation after broad-spectrum HDACi treatment, we did not observe changes in ERK activity. 

Considering the critical role of autophagy in tumor progression and tumor cell survival, treatment with targeted therapies that induce cytoprotective autophagy might inadvertently contribute to therapeutic resistance and tumor growth (reviewed in [22]). HDACi-mediated autophagy indeed has been linked to therapeutic resistance in malignant peripheral nerve sheath tumors (MPNST), where a subset of HDACi resistant cells displayed morphologic features of autophagy but not apoptosis [37]. Despite all evidence that HDACis are promising anti-cancer targets, only a few broad-spectrum inhibitors have been approved for clinical use as of now, mostly because HDACi monotherapies displayed limited success in clinical trials [62]. To improve the antitumor effects of HDACi further, great potential is seen in the combination of HDACi with other drugs. For example, disruption of HDACi-mediated autophagy by combining HDACis with drugs that inhibit late stages of autophagy, such as chloroquine or bafilomycin A1, can enhance HDACi-mediated cell death in vitro and in vivo [37,38,39]. Likewise, combination of vorinostat or panobinostat with chloroquine in our neuroblastoma model significantly reduced colony formation and cell viability of tumor cells compared to single agent treatment. This is supported by our own in vivo data from a zebrafish early larva xenograft model, where the combination treatment with panobinostat and chloroquine substantially decreased tumor volume.

In conclusion, we demonstrate that the broad-spectrum HDACis vorinostat and panobinostat induce autophagy in neuroblastoma cells on a transcriptional level. This process is regulated by the dephosphorylation and translocation of transcription factors FOXO1 and FOXO3a and occurs independently of other autophagy inducing pathways such as mTORC signaling or activation of the transcription factor TFEB. By combining HDACi with the late-stage autophagy inhibitor chloroquine, we successfully sensitized neuroblastoma cells to HDACi treatment both in vitro and in vivo. We infer that this drug combination represents a promising approach for the treatment of high-risk neuroblastomas depending on autophagy as treatment resistance mechanism.

## Data Availability

Gene expression data is available through Gene Expression Omnibus (GEO) database, accession no. GSE—will be provided during revision.

## Supplementary Materials

The following are available online at https://www.mdpi.com/article/10.3390/cells10051001/s1, Supplementary Methods: Source Code for quantification of microscopic images, Figure S1: Treatment with broad-spectrum HDAC inhibitors induces autophagic flux, Figure S2: Confocal fluorescence microscopy analysis of autophagic flux. Figure S3: Broad-spectrum HDAC inhibitor treatment activates FOXO1/FOXO3 transcription factors.
