# Supplementary material for: Broad-Spectrum HDAC Inhibitors Promote Autophagy through FOXO Transcription Factors in Neuroblastoma
Source: Cells. 2021 Apr 24;10(5):1001. doi: 10.3390/cells10051001 (PMC8144997; doi:10.3390/cells10051001)
Supplement: Supplementary file 1 [file cells-10-01001-s001.zip › Suppl Material_revised_2.docx]

## Supplementary material

Broad-spectrum HDAC inhibitors promote autophagy through FOXO transcription factors in neuroblastoma

Katharina Körholz^1, 2^, Johannes Ridinger^1, 2^, Damir Krunic^3^, Sara Najafi^1, 2, 4^, Xenia F. Gerloff^1,2,4^, Karen Frese^5^, Benjamin Meder^5,6^, Heike Peterziel^1, 2^, Silvia Vega Rubin de Celis^7^, Olaf Witt^1, 2, 4^ and Ina Oehme^1, 2,^*

^1^ Hopp Children’s Cancer Center Heidelberg (KiTZ), 69120 Heidelberg, Germany

^2^ Clinical Cooperation Unit Pediatric Oncology, German Cancer Research Center (DKFZ), INF 280, 69120 Heidelberg, Germany and German Cancer Research Consortium (DKTK)

^3^ Light Microscopy Facility (LMF), German Cancer Research Center (DKFZ), Heidelberg, Germany

^4^ Department of Pediatric Oncology, Hematology and Immunology, University Hospital Heidelberg, Heidelberg, Germany

^5^Institute for Cardiomyopathies Heidelberg, Heidelberg University, 69120 Heidelberg, Germany

^6^Genome Technology Center, Stanford University, Stanford, CA 94304, USA

^7^Institute for Cell Biology (IFZ), University Hospital Essen, Essen, Germany

*Correspondence: i.oehme@kitz-heidelberg.de

**Supplementary Figures**

**
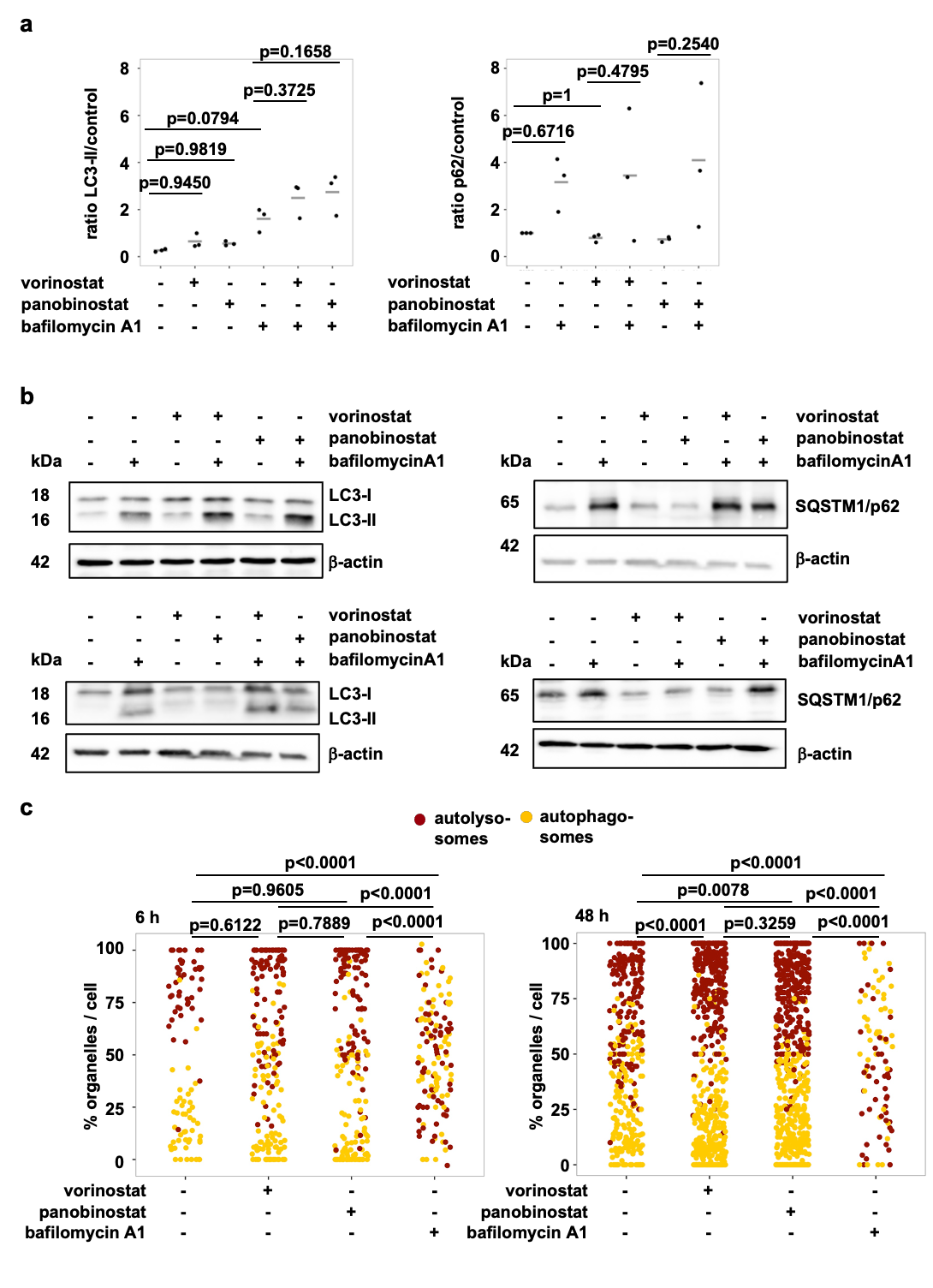
**

**Figure S1.** Treatment with broad-spectrum HDAC inhibitors induces autophagic flux. (**a**) Quantification of Western blot analysis of LC3-II and p62 expression after treatment for 24 h with vorinostat (500 nM) or panobinostat (10 nM). (**b**) Western Blot displaying LC3-I and LC3-II levels and SQSTM1/p62 levels in whole-cell lysates of SK-N-BE(2)-C cells after 24h treatment with 500 nM vorinostat or 10 nM panobinostat or solvent control and with or without additional 6h treatment with bafilomycin A1 (100 nM). Beta-actin and HSC70 served as a loading control for corresponding Western blots. (**c**) Single cell analysis of mean diagram presented in Figure 1. Automated quantification of one representative fluorescence microscopic experiments using the mCherry-EGFP-LC3B expression construct 6h and 48h after treatment with either HDACi or bafilomycinA1. Each transfected cell was analyzed for green and red fluorescent foci. Statistical analyses: ANOVA with Tukey’s multiple comparison test.


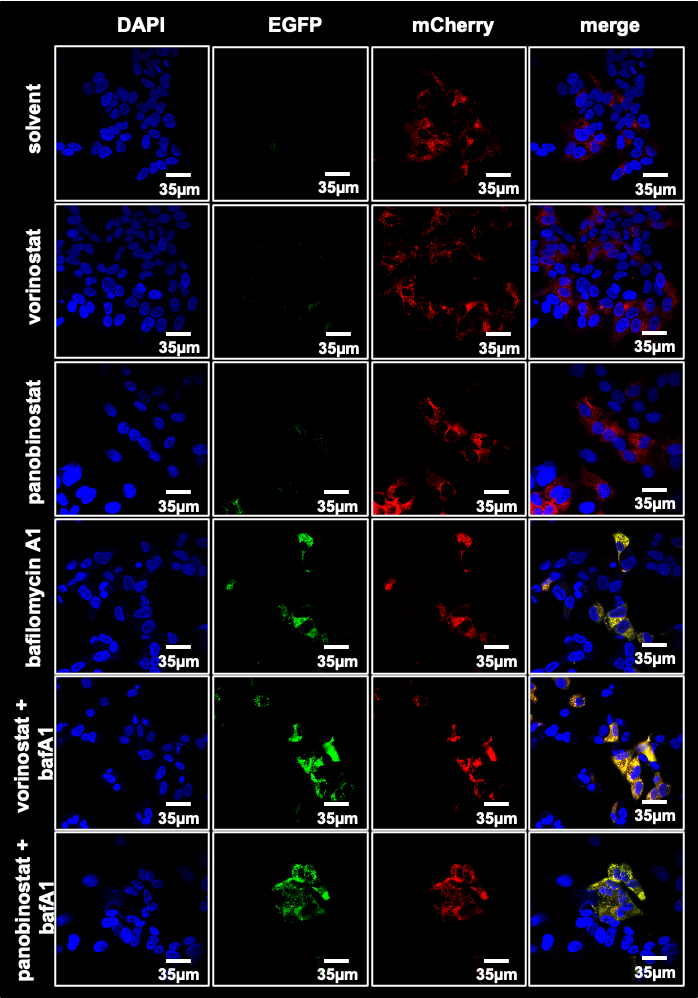


**Figure S2.** Confocal fluorescence microscopy analysis of autophagic flux. Confocal fluorescence microscopy analysis of SK-N-BE(2)-C cells. Autophagosome formation was visualized after treatment with vorinostat (500 nM) or panobinostat (10 nM) alone or in in combination with bafilomycin A1 (100 nM) by using the mCherry-EGFP-LC3B expression construct. Autophagolysosomes appear red fluorescent only. Autophagosomes appear red and green fluorescent (yellow in merged channels). Scale bar: 35 µm.


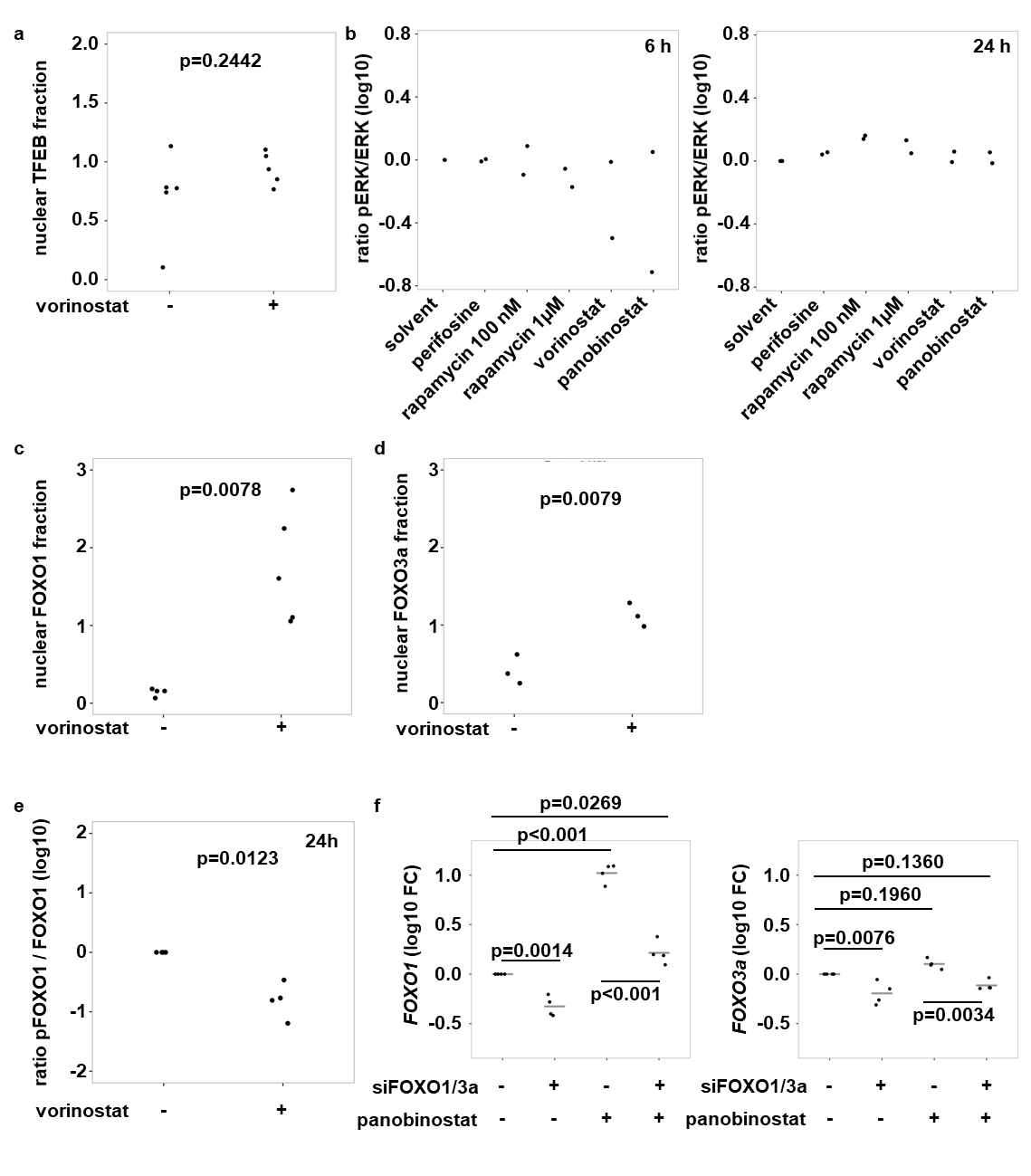


**Figure S3.** Broad-spectrum HDAC inhibitor treatment activates FOXO1/FOXO3 transcription factors. (**a**) Quantification of nuclear TFEB by Western blot analysis from subcellular localization analysis. (**b**) Quantification of Western blot analysis of pERK1/2 and ERK1/2 expression after 6h and 24h treatment with vorinostat (500 nM) or panobinostat (10 nM). (**c**–**d**) Quantification of FOXO1 (**b**) and FOXO3a (**c**) nuclear protein of at least three individual western blots. (**e**) Quantification of the pFOXO1 to FOXO1 ratio from Western blot analysis from four individual experiments, normalized to solvent control. (**f**) Realtime RT-PCR analysis of *FOXO1* and *FOXO3a* after transfection of SK-N-BE(2)-C neuroblastoma cells with control or FOXO1 and FOXO3a siRNA (pool of 3 siRNAs for each target; pool of 6 siRNAs in total). Cells were additionally treated for the last 24h with either panobinostat or a solvent control. Statistical analyses: ANOVA with Tukey’s multiple comparison test. (**a**–**e**) Statistical analyses: t-test.


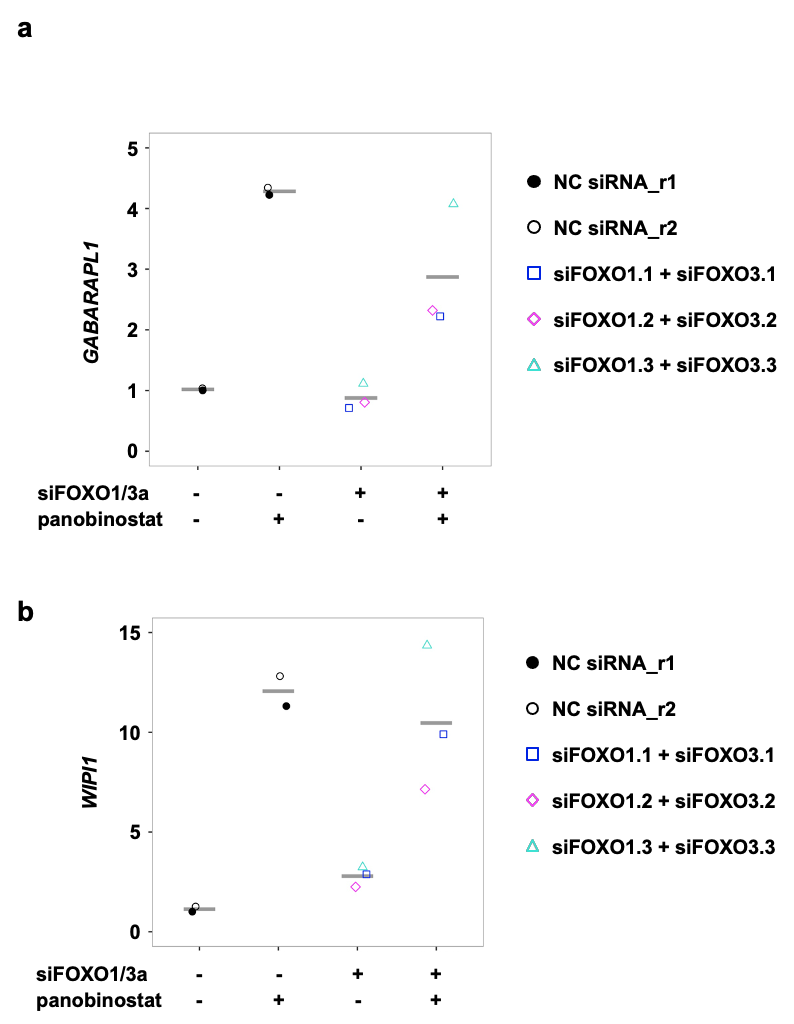


**Figure S4.** Target gene expression. Realtime RT-PCR analysis of *GABARAPL1* (**a**) and *WIPI1* (**b**) after transfection of SK-N-BE(2)-C neuroblastoma cells with control (black and open circle) or FOXO1 and FOXO3a siRNA in single siRNA combinations; blue square: siRNA1 against FOXO1 plus siRNA1 plus FOXO3a; magenta diamond: siRNA2 against FOXO1 plus siRNA2 plus FOXO3a; turquois triangle: siRNA3 against FOXO1 plus siRNA3 plus FOXO3a. Cells were additionally treated for the last 24h with either panobinostat or a solvent control.

**Suppl. Methods**

**Source Code for quantification of microscopic images**

dir = getDirectory("Choose an INPUT Directory ");

folder = getDirectory("Choose an OUTPUT Directory");

list = getFileList(dir);

roiManager("Reset");

run("Close All");

DAPI=getNumber("Ch. for DAPI", 1);

RED=getNumber("Ch. for RED", 3);

GREEN=getNumber("Ch. for GREEN", 2);

minsize=getNumber("Min. size of particles", 5);

minnoise=getNumber("Min. noise for Find Max.", 1000);

for (i=0; i<list.length; i++) {

showProgress(i, list.length);

open(dir+list[i]);

bit=bitDepth();

if (bit==8) max=255;

if (bit==16) max=65535;

title= getTitle();

image= folder+title;

roiManager("Reset");

//segment nuclei

run("Set Scale...", "distance=0 known=0 pixel=1 unit=pixel global");

setSlice(DAPI);

run("Duplicate...", " ");

run("Subtract Background...", "rolling=50");

run("Median...", "radius=2");

setAutoThreshold("Default dark");

run("Threshold...");

setThreshold(8000, max);//change Thr. to get more / less nuclei

waitForUser("Please check the Thr.");

wait(500);

setOption("BlackBackground", true);

run("Convert to Mask");

run("Fill Holes");

run("Watershed");

run("Analyze Particles...", "size=2000-Infinity summarize add");//change size to get more / less nuclei

roiManager("Save", image+"Nuclei.zip");

close();

//segment RED cells

setSlice(RED);

run("Duplicate...", " ");

run("Subtract Background...", "rolling=100");

setForegroundColor(255, 255, 255);

run("Median...", "radius=5");

run("Threshold...");

setThreshold(1800, max);

run("Grays");

waitForUser("Please check the Thr.");

wait(500);

//change Thr. to get more / less RED cells

run("Create Selection");

roiManager("Reset");

roiManager("Add");

roiManager("Save", image+"Cells.zip");

roiManager("Reset");

run("Select None");///////////

roiManager("Open", image+"Nuclei.zip");

makePoint(18, 22);

roiManager("Add");

roiManager("Combine");

setMinAndMax(0, max);

run("8-bit");

roiManager("Fill");

roiManager("Reset");

roiManager("Open", image+"Cells.zip");

roiManager("Select", 0);

setBackgroundColor(0, 0, 0);

run("Clear Outside");

setThreshold(1, max);

run("Find Maxima...", "noise=55 output=[Segmented Particles] above");//change to get more / less RED cells

roiManager("Reset");

rename("RED cells.tif");

run("Analyze Particles...", "size=2000-Infinity summarize add");//change size to get more / less RED cells

close();

close();

run("Make Composite");

roiManager("Show None");

roiManager("Show All");

waitForUser("Please check the ROIs");

print(title+" "+roiManager("Count") + " RED cells corr.");

wait(500);

run("Select None");

roiManager("Deselect");

roiManager("Save", image+"Individual_Cells.zip");

roiManager("Reset");

//prepare for RED/GREEN segmentation

setSlice(GREEN);

run("Duplicate...", "title=GREEN channels=1-3");

selectWindow(title);

setSlice(RED);

run("Duplicate...", "title=RED channels=1-3");

//segment RED

resetMinAndMax();

run("Subtract Background...", "rolling=3");

run("Despeckle");

run("Threshold...");

setThreshold(1000, max);

run("Grays");

waitForUser("Please check the Thr. for RED");

wait(500);

run("Find Maxima...", "noise="+minnoise+" output=[Segmented Particles] above");

//segment GREEN

selectWindow("GREEN");

run("Subtract Background...", "rolling=3");

run("Despeckle");

run("Threshold...");

setThreshold(1000, max);

run("Grays");

waitForUser("Please check the Thr for GREEN");

wait(500);

run("Find Maxima...", "noise="+minnoise+" output=[Segmented Particles] above");

selectWindow("RED Segmented");

saveAs("Tiff", folder+"RED Segmented.tif");

selectWindow("GREEN Segmented");

saveAs("Tiff", folder+"GREEN Segmented.tif");

run("Close All");

//analyze COLOC

open(folder+"RED Segmented.tif");

open(folder+"GREEN Segmented.tif");

r="RED Segmented.tif";

g="GREEN Segmented.tif";

run("Colocalization ", "channel1=["+r+"] channel2=["+g+"] ratio=[0] threshold=[14] threshold=[14] display=255 also");

//to exclude GREEN without RED (w/o coloc)

roiManager("Open", image+"Individual_Cells.zip");

roiManager("Combine");

run("Clear Outside");

roiManager("Reset");

run("Select None");

run("Split Channels");

selectWindow("Colocalizated points (RGB) (green)");

rename("green");

gr="green";

run("Subtract...", "value=100");

selectWindow("Colocalizated points (RGB) (blue)");

rename("blue");

bl="blue";

re="*None*";

run("Analyze Particles...", "size="+minsize+"-Infinity add");

roiManager("Combine");

run("Clear Outside");

roiManager("Reset");

run("Select None");

run("Merge Channels...", "c1="+re+" c2="+gr+" c3="+bl+"");

run("8-bit");

setThreshold(25, 255);

run("Find Maxima...", "noise=100 output=[Segmented Particles] above");

setAutoThreshold("Default dark");

run("Create Selection");

roiManager("Add");

close();

close();

close();

selectWindow("GREEN Segmented.tif");

wait(200);

roiManager("Select", 0);

run("Clear Outside");

roiManager("Reset");

run("Select None");

roiManager("Open", image+"Individual_Cells.zip");

NRoi=roiManager("Count");

for(k=0; k<NRoi; k++)

{

rename(title+" GREEN Cell Nr"+k+1);

roiManager("Select", k);

run("Analyze Particles...", "size="+minsize+"-100 summarize add");//check min size...

}

roiManager("Save", image+"Individual_Cells with GREEN.zip");

roiManager("Reset");

roiManager("Open", image+"Individual_Cells.zip");

close();

selectWindow("Colocalizated points (8-bit) ");

rename(title+" Coloc");

for(k=0; k<NRoi; k++)

{

rename(title+" Coloc Cell Nr"+k+1);

roiManager("Select", k);

run("Analyze Particles...", "size="+minsize+"-100 summarize add");//check min size...

}

roiManager("Save", image+"Individual_Cells with COLOC.zip");

roiManager("Reset");

roiManager("Open", image+"Individual_Cells.zip");

close();

//analyze RED and GREEN

//selectWindow("RED Segmented.tif");

//open(folder+"RED Segmented.tif");

selectWindow("RED Segmented.tif");

for(k=0; k<NRoi; k++)

{

rename(title+" RED Cell Nr"+k+1);

roiManager("Select", k);

run("Analyze Particles...", "size="+minsize+"-100 summarize add");//check min size...

}

roiManager("Save", image+"Individual_Cells with RED.zip");

roiManager("Reset");

run("Close All");

}
