# Supplementary material for: Broad-Spectrum HDAC Inhibitors Promote Autophagy through FOXO Transcription Factors in Neuroblastoma
Source: Cells. 2021 Apr 24;10(5):1001. doi: 10.3390/cells10051001 (PMC8144997; doi:10.3390/cells10051001)
Supplement: Supplementary file 1 [file cells-10-01001-s001.zip › SupplFig2_neu_rev2.pdf]

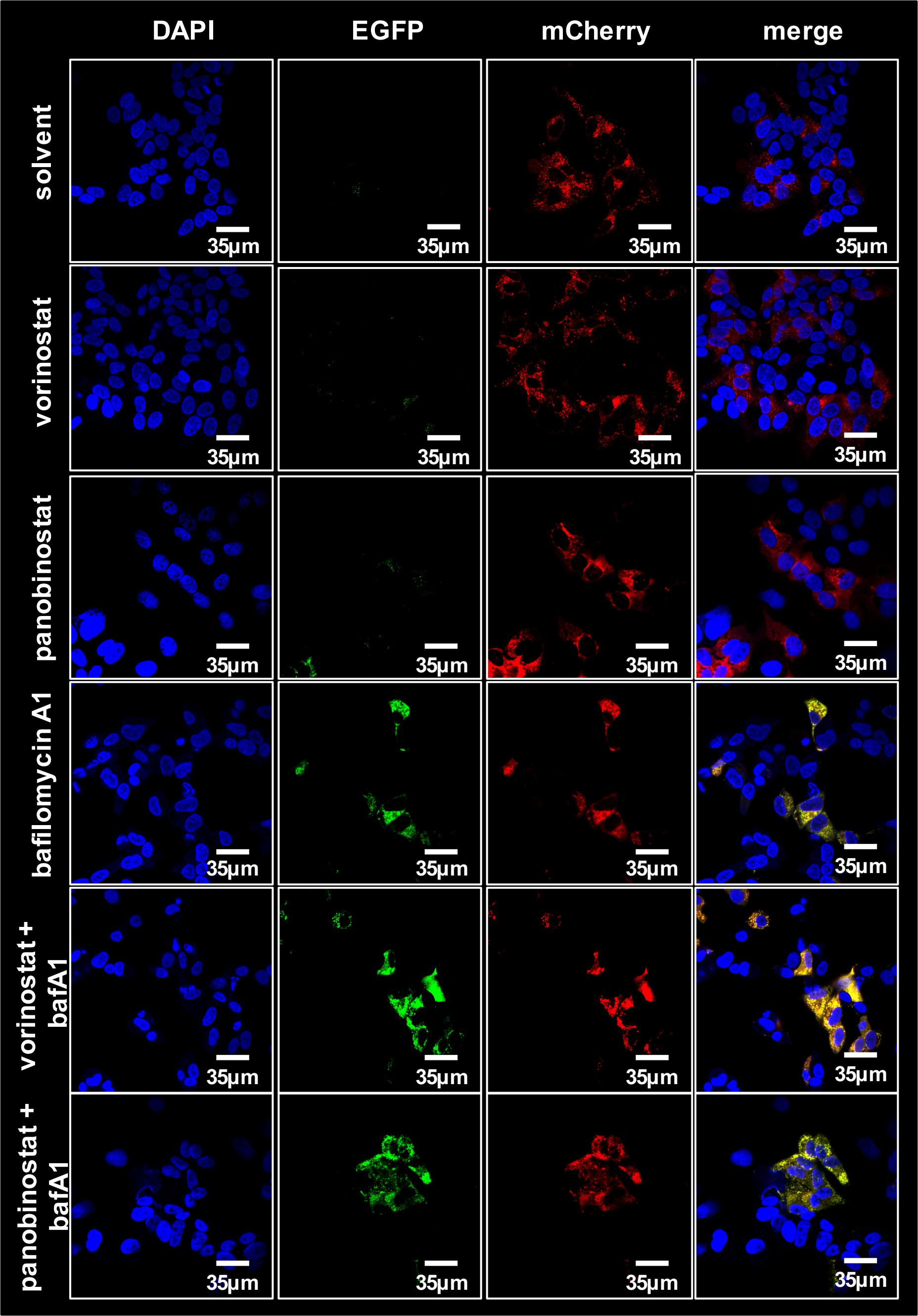

DAPI
EGFP
mCherry
merge
solvent
vorinostat
panobinostat
bafilomycin A1
vorinostat + bafA1
panobinostat + bafA1
35µm
