# Supplementary figures and images for: Broad-Spectrum HDAC Inhibitors Promote Autophagy through FOXO Transcription Factors in Neuroblastoma
Source: Cells. 2021 Apr 24;10(5):1001. doi: 10.3390/cells10051001 (PMC8144997; doi:10.3390/cells10051001)

**a**

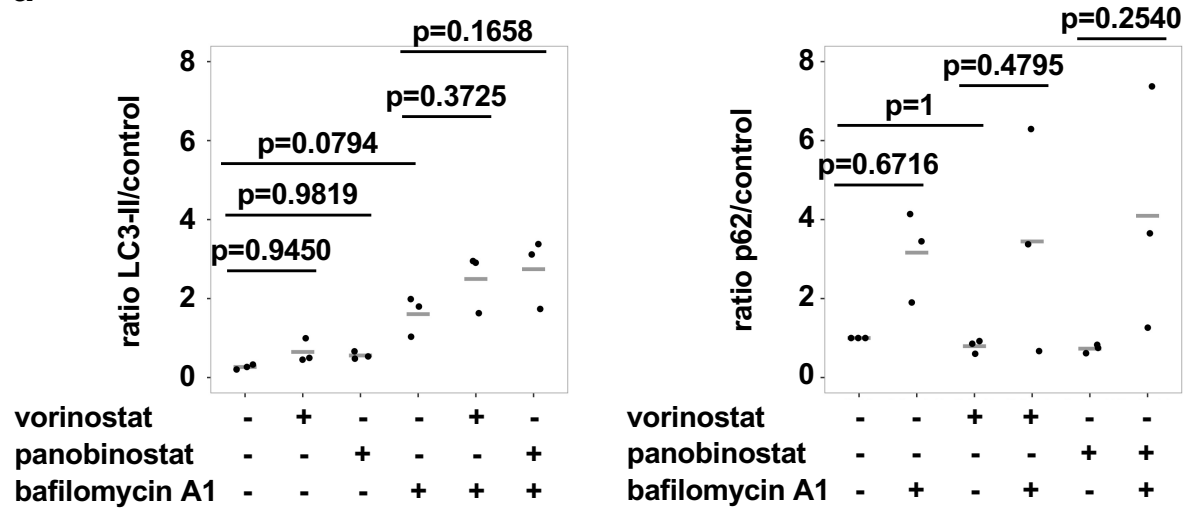

Supplemental Fig. 1

**b**

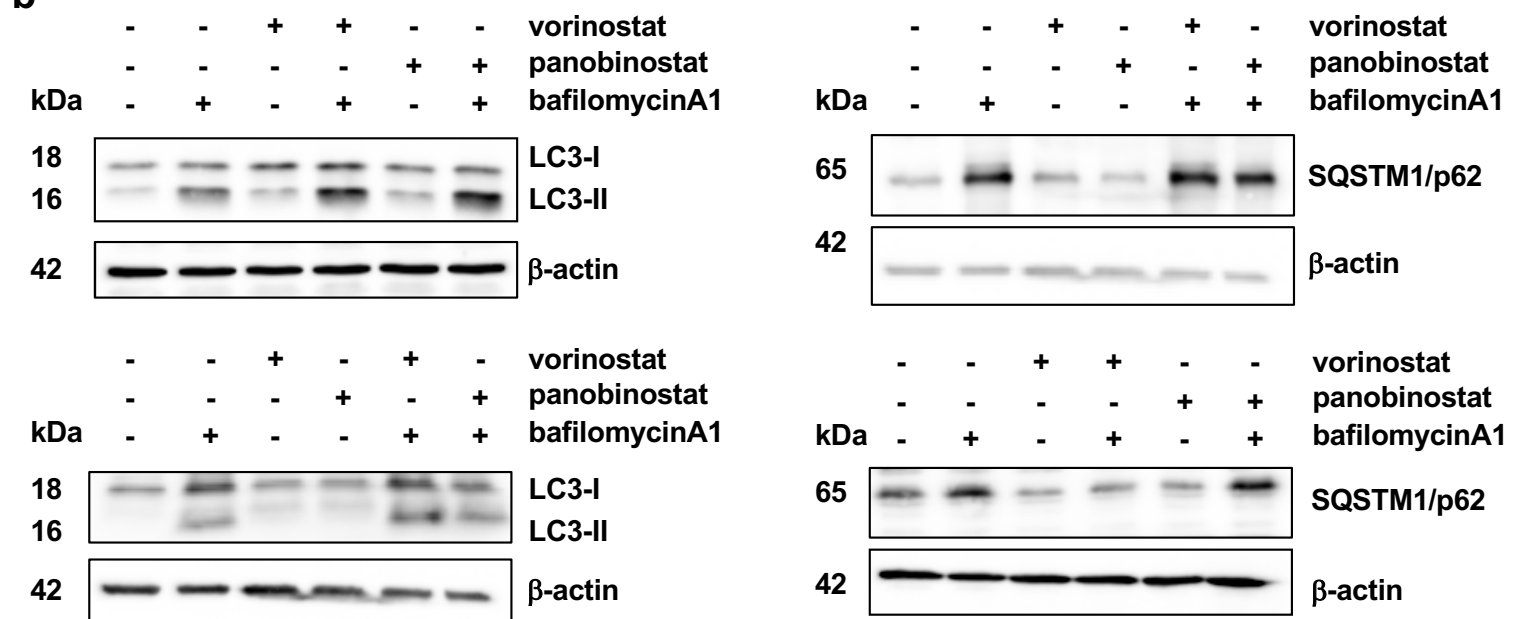

**c**

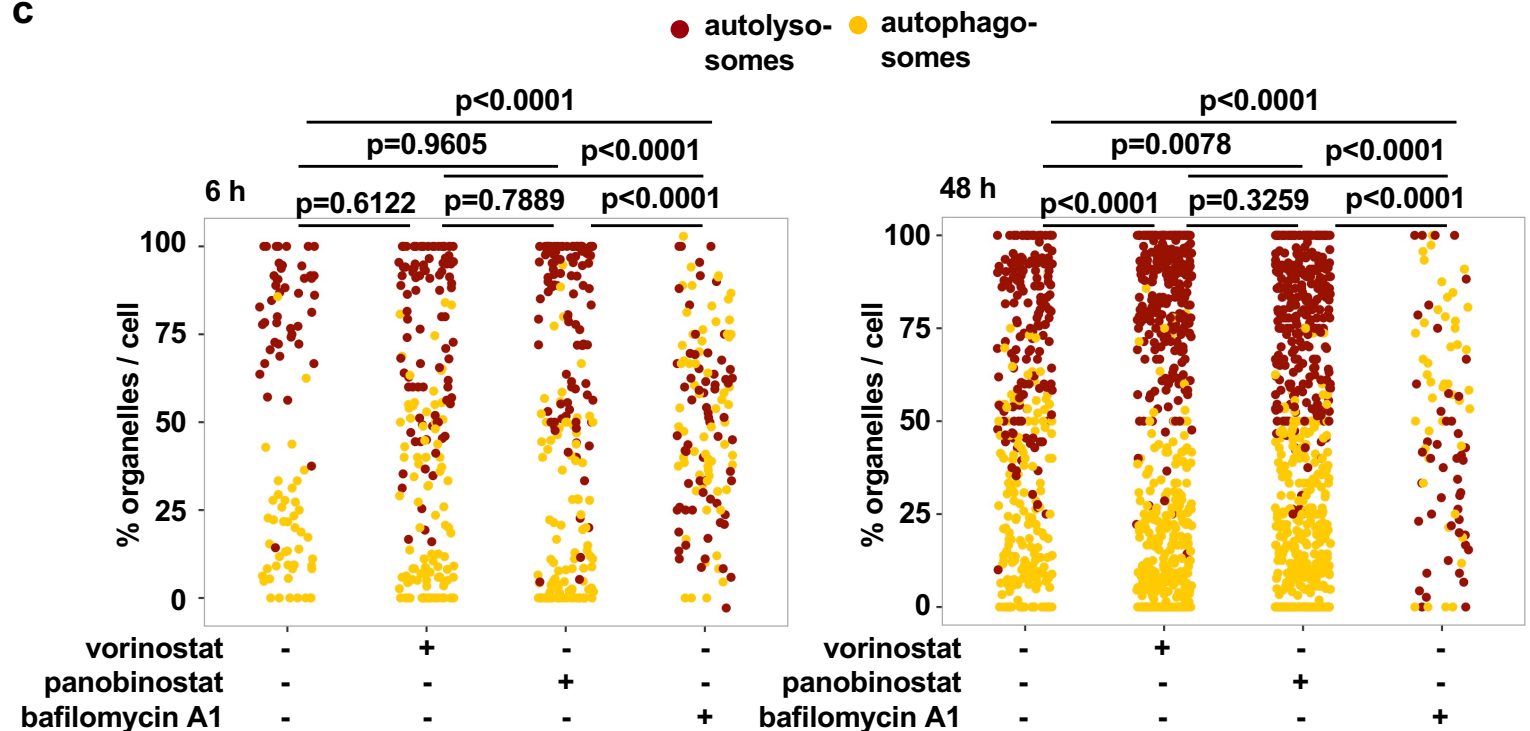

Supplement: Supplementary file 1 [file cells-10-01001-s001.zip › SupplFig1_neu_rev2.pdf]

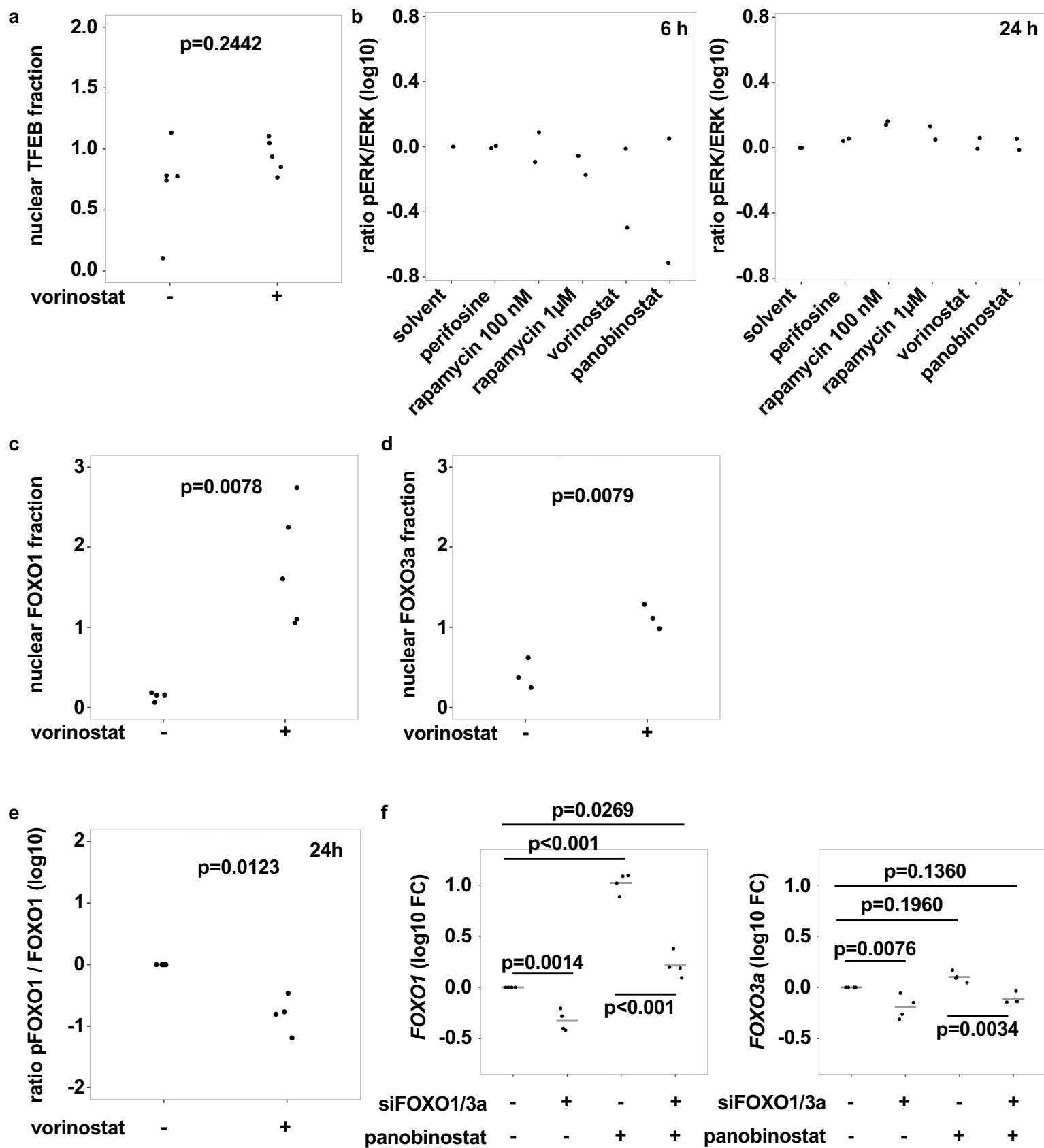

Supplement: Supplementary file 1 [file cells-10-01001-s001.zip › SupplFig3_rev2.pdf]

**a**

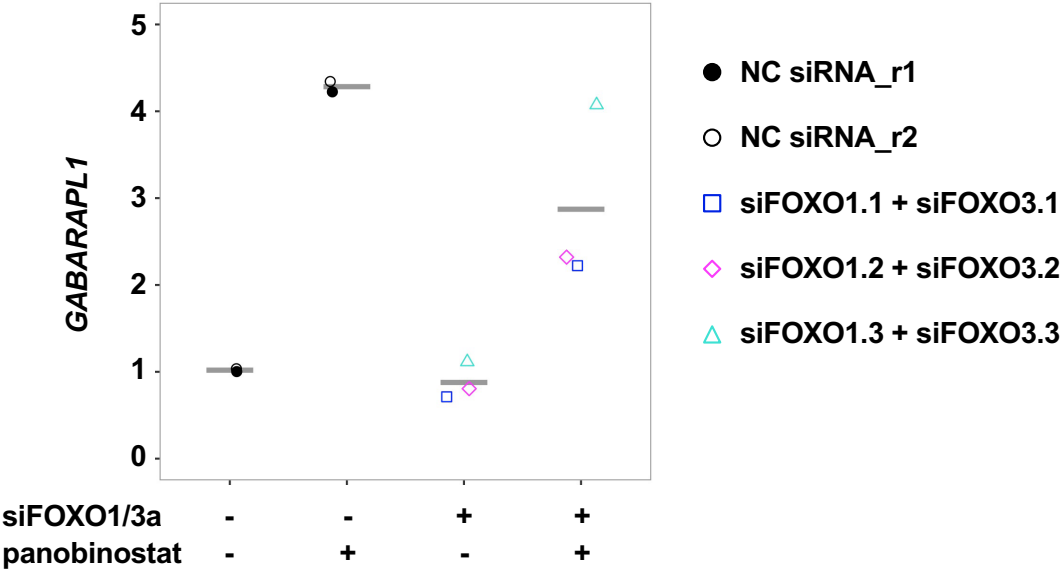

**b**

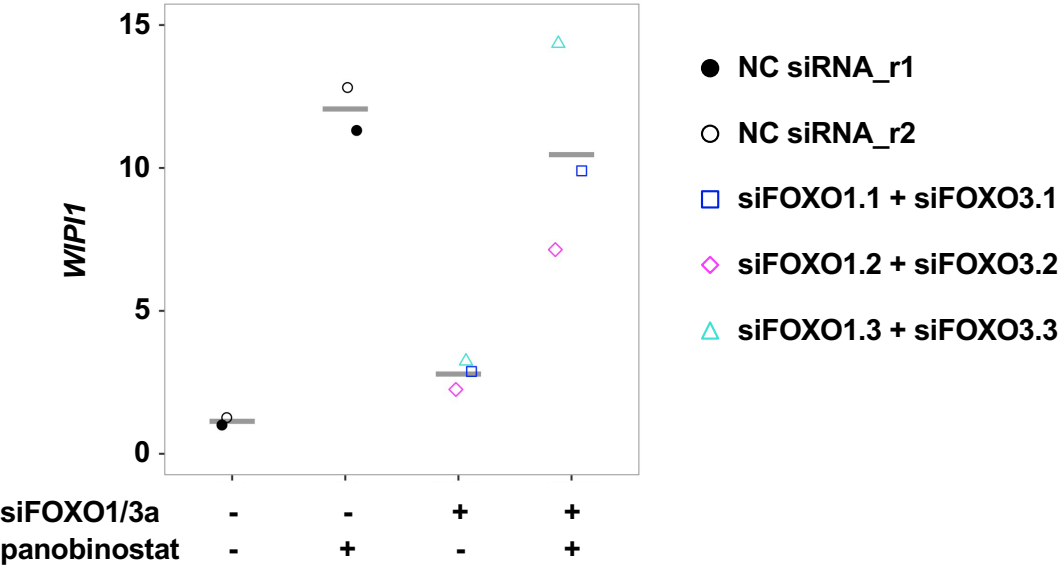

Supplement: Supplementary file 1 [file cells-10-01001-s001.zip › SupplFig4_rev2.pdf]
